# **Experiences of participating in cortisol awakening response research:***“I was more consciousthan usual, I wanted to get it right”*

**DOI:** 10.1016/j.cpnec.2024.100276

**Published:** 2024-11-06

**Authors:** Natasha Ramachandran, Nina Smyth, Sanjay Joban, Maria Flynn, Angela Clow, Lisa Thorn

**Affiliations:** School of Social Sciences, University of Westminster, London, UK

**Keywords:** Awakening, Ecological validity, Salivary cortisol, Qualitative research, Participant experience, CAR methodology

## Abstract

Cortisol awakening response (CAR) research relies upon self-collected saliva sampling during the post-awakening period. It is unknown how the CAR protocol is perceived and how they may affect typical routines relevant to CAR methodology. CAR assessment is sensitive to state variables, suggesting that CAR measurement may be affected by research participation. This is the first qualitative study to explore motivation and experiences of participation in CAR research.

Interviews were conducted with a sample of 20 participants (males/females: 4/16) aged 46-82 years following their participation in CAR research in the domestic setting. Responses were transcribed verbatim and thematically analysed.

Participants were motivated to take part in CAR research for altruistic reasons and the apparent convenience of undertaking the study at home. Participants experienced the study as arduous describing apprehension and the cognitive burden it placed on them leading to disruptions to sleep and morning routines. Participants also struggled to identify the moment of awakening and there was uncertainty surrounding the timing of the first awakening sample. Disruptions were lessened with habituation to sampling on repeated study days.

There was apprehension about taking part in CAR research, affecting mood, cognition, and sleep; state variables known to influence the CAR. Findings inform ways to optimise CAR ‘ecological validity’ and obtain typical CAR characteristics. The ‘moment of awakening’, was not universally understood, leading to hesitancy in deciding when to collect saliva samples. Researchers need to include a specific discussion of the commonly experienced ambiguity surrounding awakening to increase awareness, lessen anxiety and highlight its importance.

## Highlights

• Participation in CAR research increased apprehension for participants.
• Participation in CAR research impacted mood, cognition and sleep.
• Participant apprehension habituated with repeated sampling days.
• Participants reported difficulty identifying the moment of awakening.
• Suggestions supplement the CAR Consensus papers (Stalder et al., 2016, 2022).

## 1 Introduction

The cortisol awakening response (CAR) is the burst of cortisol secretion following awakening from night-time sleep and can be measured in groups of humans from saliva samples [[1], [2], [3]]. The CAR is a prevalent measure in psychoneuroendocrinology research, and it is associated with trait measures, such as cognitive function, in both clinical and healthy populations [[4], [5], [6], [7]]. The CAR is typically measured from volunteers who agree to collect their saliva samples at home, which is credited with providing ‘ecological validity’ i.e., insight into CAR characteristics unaffected by the demands of overnight attendance at sleep laboratories and direct researcher oversight. However, there are methodological issues in CAR assessment resultant from marked intra-individual variability accounting for between 61 % and 82 % of its measurement [[8], [9], [10]]. These day-to-day differences are associated with state variables e.g., prior day mood, anticipation of the day ahead and time of awakening [11], variables that are potentially affected by study participation which may exacerbate the problem.

Another methodological issue in CAR research is associated with sampling accuracy to detailed timing protocols of saliva samples in the immediate post-awakening period. This has led to concern about the robustness of many published studies [11,12] and can be due to delays in collecting saliva samples. Delays may be due to non-conscientious participant behaviour, or participants not being aware of the exact timing of waking, likely caused by the immediate post awakening period being associated with a reduced state of cognitive and motor performance, resultant from sleep inertia [13]. This may increase the difficulty of accuracy to the saliva sampling protocol, even in the most painstaking and conscientious participants [14,15] These issues are important as even short delays between awakening and the commencement of sampling impact CAR measurement [11,12,16].

Accurate measurement of the CAR requires participants to be aware of the ‘moment of their awakening’ and commence sampling immediately afterwards [11,12]. Inability to identify the moment of awakening would contribute to sampling inaccuracy by increasing delay in initiating saliva sampling. Clearly accurate CAR measurement relies upon participants identifying their ‘moment of awakening’ as well as adherence to the strict timing protocol following awakening. The CAR consensus guidelines [11,12] advise engagement with participants at the point of recruitment to ensure the highest levels of adherence to the required protocol. The guidelines emphasise the need to identify the ‘moment of awakening’ as the starting point for the initiation of saliva sampling. The onus to explain precisely what is meant by ‘the ‘moment of awakening’ to the participant is on the researcher during the initial face-to-face or telephone briefing [11,12]. The definition in the consensus paper, focuses on the ‘regaining of consciousness’. Stalder [11] explains:

**“** *When you are awake, i.e. you are conscious: you know who and where you are; you are in a state that clearly is different from when you were sleeping even though you may still feel tired ….”*

It is unknown how participants interpret these instructions and what impact the study demands may have on their psychological state, typical sleep patterns and awakening routines. The purpose of this study was to use in-depth one-to-one interviews to explore motivations to volunteer for CAR studies and the subsequent experience of being a participant with the aim of informing best practice research methodology.

## 2 Method

This study employed qualitative analysis of interview data using a thematic analysis approach to better understand participants’ overall experiences of participating in CAR research. The study was approved by the Research Ethics Committee at the University of Westminster (Ethics reference: ETH1819-0331, ETH 1516-1354).

### 2.1 Participants

Participants (N = 20) were sampled from two populations, both of whom had recently participated in CAR research (see Table 1). Participants with primary open-angle glaucoma (POAG, N = 12) were recruited from the International Glaucoma Association (IGA). These participants had taken part in a study examining the CAR with primary open-angle glaucoma. Ages ranged between 62 and 82 years, and all were retired. Across all days, verified median wake time (determined by actigraphy) for those with POAG was 06:44 hh:mm (interquartile range: IQR 5:35–07:05). Wake time determined by self -report for those with POAG was 06:43 hh:mm (interquartile range: IQR 5:35–7:05). Participants with POAG were highly accurate when completing the sampling protocol with a medium interval of 0 min (IQR: 0-1 min) between awakening and collection of the first morning sample.,

**Table 1:** Demographics of participants, sampling days and interview methods.

Sample	Age (years)	Gender	CAR sampling days	Interview method
Glaucoma (N = 12)	M = 70.1 SD = 6.1	Male = 2 Female = 10	2 consecutive midweek days	FF = 1 T = 10
Academic (N = 8)	M = 50.7 SD = 2.2	Male = 4 Female = 4	4 midweek days, one week apart	FF = 9
Total	M = 61.2 SD = 10.9	Male = 6 Female = 14		FF = 10 T = 10

FF = Face-to-face, T = Telephone.

A second group of participants were members of the academic community at the University of Westminster (N = 8) who took part in a study exploring the relationship between the CAR and visual dependency in postural control [17]. Ages ranged between 48 and 55 years old. Across all days, verified median wake time (determined by actigraphy) for members of the academic community was 06:01 hh:mm (interquartile range: IQR: 05:59–06:49 hh:mm). Participants were also accurate to the saliva sampling protocol, with a median interval of 2 min (IQR = 1–3 min) between awakening and collection of the awakening sample (data reported from Ref. [17]. All participants self-reported that they were in good health.

### 2.2 Data collection

Face-to-face and telephone semi-structured interviews were conducted. Participants provided informed written consent prior to the study. The interviewers re-established consent verbally at the commencement of the interview, checking participants wished to proceed and reiterated their right to decline to answer questions and to stop the interview at any time. A semi-structured interview guide facilitated conversations around motivation and experience of taking part in CAR research (See Table 2 for indicative questions). Interviews lasted 30 min on average and were conducted within a week of the final saliva sampling day for each study by two experienced qualitative researchers (SJ and NR). All interviews were audio-recorded and then transcribed verbatim by NR.

**Table 2:** Indicative interview topic guide.

Topic
What were your reasons for taking part in this study? What were your general feelings about participation? (e.g., providing saliva samples upon waking up?) How did your routine/experience compare to your usual routine? Did you do anything differently? What was the experience like for you to collect the saliva samples over the two mornings? What was it like collecting samples as soon as you woke? How did you determine this ‘moment of awakening’ as requested by the team? At what point, after gaining consciousness, do you normally feel fully alert? How would you describe your awaking process? What do you go through as you are waking up?

### 2.3 Data analysis

The interview data was analysed using the recommended thematic analysis framework [18]. The phases included familiarisation with the data, generating initial codes, searching for themes, reviewing themes, and defining themes. Three transcripts were analysed independently (by SJ, NR, and LT) and coding was discussed until consistency was reached. Two authors (NR and SJ) analysed the remaining transcripts independently. To increase reliability, regular analysis meetings were conducted to discuss new themes and instances of discrepancy in coding. Once overarching themes and subthemes were refined extracts from narratives that illustrated them were selected to elucidate findings. Only data relating to personal experiences of CAR research participation are presented in this paper. Excerpts from participant narratives are coded ‘G’, for those participants with POAG and ‘A’ for those recruited from among the academic community.

## 3 Results

The analysis identified eight themes grouped into three overarching themes, which encompass the aspects of participation, including motivations for taking part in the study, the experience of participation, and their understanding and identification of the moment of awakening.

### 3.1 Motivations for taking part

Participants’ motivations for putting themselves forward to participate in the CAR research studies were both extrinsic and intrinsic, illustrated by a sense of altruism and the perceived convenience of the study. Data indicated that these participants were highly motivated, despite receiving no financial incentive to take part in the CAR studies or in the current study.

#### 3.1.1 Altruism

Participants expressed intrinsic altruistic motivations which were linked to the perception of research as worthwhile. These sentiments were directed towards research in general, as well as towards the researchers, and the specific CAR study in which they participated. Participants expressed little self-interest going so far as to characterise their decision to participate from a sense of social obligation: “*I feel a sort of ‘duty’, but [it] comes off a bit strong, a duty to do our bit. If [I] can be of any help, even if it helps you. I have taken part in medical trials, and I feel it is worth doing!*” (P08, G). Participants voiced a wish to help further knowledge alongside a general sense of wanting to benefit others: “*Since I was diagnosed with glaucoma, I'm happy to do anything that I can to shed any light that may help with it all. I also like to help anyone else that would benefit*” (P11, G). Altruistic motivation was linked to promoting awareness and dissemination of a particular condition to “*help out and get that knowledge out there*” (P03, G).

Similarly, members of the academic community at the University voiced a motivating awareness of ethical responsibility to participate in research to gain insight into the participants' experiences, given that they also recruit participants for studies. For example, P15 (A), an experienced researcher has conducted several studies in the past explained, sympathised with researchers, and explained: *I guess because I sympathise with people trying to recruit participants; so that's the first thing, is that I wanted to be able to be helpful”* (P15, A).

#### 3.1.2 Apparent convenience

The CAR research in which these participants were involved took place in the domestic setting, saliva sampling packs were posted or collected at their convenience. Taking part in the study in the comfort of one's own home without the necessity of travel was an important motivator for participants: *“I didn't have to leave the home to do it. I could do it all in my own home”* (P01, G). Participants with glaucoma expressed their eagerness to participate in research studies but that the nature of those studies was often prohibitive: *“Well, I had glaucoma for quite a few years now and it has got worse. I did apply once before to the glaucoma magazine [to take part in a study]. They wanted me to go to London three times, I think. There was no way I could do that. And so, when this came up, I thought oh that's interesting and that's something I could do”* (P05, G). Prior to participation there was a general perception that saliva sampling at home would be relatively uncomplicated, and participants reported feeling confident in the protocol: “*Oh well, I mean it all seemed very straightforward, non-invasive, not a problem”* (P07, G).

Others from the academic community, were very vocal with the overall convenience they felt with the sampling protocol. Through prior participation, one participant was aware of the guidelines which required the participant to not doze or fall asleep between saliva sampling times. Due to the nature of their own sleeping and waking habits they found the protocol easy to fit into their waking routine as they explained that they usually stay awake once awake. P18 (A) states: “*Once I kind of wake up, I usually [stay] up …. I might listen to the radio – just lie and listen to the radio or something like that – for half an hour … so that … so in that sense … doing the samples was … was relatively easy*”.

### 3.2 Experiences of taking part

Despite remarks from some participants about the perceived straightforwardness of the instructions and apparent simplicity of sampling in the domestic setting, narratives revealed that participation in CAR research was experienced as relatively demanding. Participants reported increased apprehension and cognitive burden. They also experienced disruptions to sleep and morning routines. However, participants described an alleviation of these issues with repeated sampling days.

#### 3.2.1 Apprehension

Apprehension was prevalent throughout the narratives. Participants expressed some ‘nervousness’, ‘worry’ and ‘concern’ when asked to describe their experiences before and during the study. Most of this apprehension appeared to be anticipatory stemming from a sense of wanting to get the study protocol right. Several participants were particularly concerned that their irregular sleep patterns would be detrimental to the study protocol: *“Yeah, you know, [I] tried to visualise in my mind exactly what I had to do and what could go wrong here. And I think my biggest worry from the beginning to the end of it was the fact that I sleep so badly.”* (P02, G). As part of the protocol participants were advised to return to sleep if wake time was premature. There was an expectation from researchers that this was possible, but for some participants this may have not been. One participant explained: *“I was a bit anxious because I had spoken to you before about a very erratic sleep pattern - not regular. Some days I do wake up really early. I was never sure. I'm never sure if I can get back to sleep again. That was my concern really*” (P01, G). The combination of concern about premature awakening, returning to sleep and timing of the first waking sample created apprehension. Others framed their apprehension in terms of the commitment required for this type of study in comparison to other research: *“Yeah, I was a little bit nervous about it … It is a real commitment; it's not something where you're just filling in a questionnaire and sending it off; it did require me to be mentally prepared, and to commit myself to it.* (P15, A).

#### 3.2.2 Cognitive burden

The idea of visualisation and mental preparation illustrated in the above excerpts was a common theme in the transcripts. There was a sense of cognitive burden as participants made efforts to internalise the study protocol, with the first study day particularly requiring focus: *“I think just being mentally prepared and … you know … reminding yourself that's what you're going to be doing when you wake up [with reference to the saliva sampling protocol]”* (P15, A). Some participants further elaborated by explaining that prior to sleeping the night before the morning sampling there was concern about remembering the protocol: *“I thought that was … yeah, it actually was more … and I say, a concern, rather than anxiety, about forgetting or not putting things [electronic devices] right, and … sort of … ‘[I have] got to remember this’ and sort of like going to bed and thinking “I hope I remember properly when I wake up” and all that kind of thing, to actually do the stuff [sampling]”* (P18, A). In general, participants described being more alert than usual before and during the study, again with the aim of getting the protocol right and achieving the best outcome for the study: *“[I] think I was more conscious than usual, I wanted to get it [the study protocol] right …”* (P12, A).

#### 3.2.3 Impact on sleep

There were consistent accounts about the impact of the study on sleep in the narratives. Participants reported shortened sleep durations and earlier morning awakening than usual. A common experience was to wake before their alarm sounded on study days: *“… it's interesting that on all four occasions I actually ended up waking up before the alarm sounded; so, I think that's quite an interesting observation …. I actually got up a whole hour before I had allowed myself to wake up”* (P17, A). Narratives such as this indicated that participants experienced the study as intruding on their sleep-wake cycle. Premature awakening was often followed by uncertainty and confusion about whether to take the first morning sample. One participant noted the difficulty: *“So normally my alarm would go off at about 7am; I might wake up before it, but this [time] … I was definitely waking up, and … I guess, under normal circumstances, I would wake up and go back to sleep; but I couldn't; I didn't want to risk it [missing the first morning sample]”* (P06, A). As well as early waking there was a general sense in participants' accounts of sleep disruption during the study period: *“I seem[ed] to have a disturbed sleep pattern because … I woke up in the middle of the night … but well when I was waking up in the early morning, it was still quite dark … I felt like I was going to get up ….it was earlier [than my usual wake time]”* (P03, G).

#### 3.2.4 Disruption to post-awakening routine

Participants were encouraged to integrate the study protocol into their customary morning routine as far as possible. However, rather than being able to continue their routines, participants described disruptions and delays. The protocol requires that participants refrain from brushing their teeth and drink nothing but water during the study period and this issue was recurrent in the narratives: *“I wake up in the morning and the first thing I do is brush my teeth; that's my routine … And plug in the kettle and have a cup of tea; that's the start of my day. And so that was disrupted because I could do neither for 45 min* (P18, A). Other participants described a similar problem of disruptions to their morning activity: *“I often sort of do go jogging first thing in the morning when I get up, and that kind of … disrupts the routine then; sort of … because nearly an hour goes by …”* (P14, A). Participants with glaucoma were asked by the researchers to carry out the saliva sampling prior to their usual morning administration of glaucoma medication as it causes dry mouth. This delay was noted by these participants: “*I had to take the [eye] drops after I did the [saliva sampling] test. So that made me [begin my day] a bit later and my husband moved into another room …. we sort of came to an arrangement between us because he gets up sometimes early morning, to go to the bathroom and he said he didn't want to disturb me ….”* (P05, G). This was not the only instance of sleeping arrangements being altered to avoid interference with the study protocol. A recurrent narrative across all transcripts for both samples of participants was that their morning routine was postponed so that they could focus on the timing of the saliva samples: *“45 min I felt, for me, my life was on hold! I was a little bit ‘clock watching’ and not doing very much else, really” (*P12, A). The extent of disruption to morning routines and the attention given to the study is typified by the following quote: *“Funnily enough, now that I think about it, I didn't even get dressed until the 45 min were up! I stayed in bed for the first one [sample] and then got up then went downstairs and I don't think I got washed or dressed”* (P03, G). Although disruption to routine was a common narrative as there were also instances of the protocol fitting in with usual morning routines, “*Once I kind of wake up, I usually [stay] up …. I might listen to the radio – just lie and listen to the radio or something like that – for half an hour … so that … so in that sense … doing the samples was … was relatively easy*” (P18, A). The nature of their habitual sleeping and waking habits were conducive to the study protocol.

#### 3.2.5 Habituation in latter study days

Although some participants reported continuing issues around sleeping, waking and sampling for the duration of the study there were indications of an easing of demands of the study protocol in latter study days. Participants reported waking later: *“ …. because we only had four days; and the first two of those, it [morning wake] was markedly earlier …”* (P14, A) and improved quality of sleep: *“The first night [prior to first study day] I didn't sleep very well but the second night was much better”* (P09, A). Participants expressed an improvement in mood: *“And you know, I felt more positive in terms of [my] mood on the second day as well, so that made the sampling easier”* (P04, A) and there was also a sense of easing of the cognitive burden in subsequent study days: *“I suppose the second morning was easier than the first, because I knew what I was doing …. I got the things ready …”* (P10, A). Improvements in sleep, mood and cognitive load therefore highlight the general ease of sampling amongst participants.

### 3.3 Identifying the moment of morning awakening

A crucial aspect of the CAR protocol is that participants collect their first saliva sample on awakening. Participants' thoughts surrounding their ability to identify the moment of morning awakening generated some interesting data since, although we wake from sleep every day, we rarely reflect on this process. A single theme emerged from the data which encapsulated participants’ difficulties with thinking and talking about the experience of the transition from sleep to waking prior to collecting their first sample.

Participants' thoughts about their sleep-wake transition in general resulted in reflections characterised by lengthy pauses and expressions of the difficulty of defining awakening eliciting exclamations such as, *“It's a mystery, isn't it?” (*P11, G) and “*I've never thought ‘how do I wake up?’*” (P20, A). Descriptions focussed on awakening as a state of consciousness, for example, *“it's consciousness, something is moving and you're becoming aware of things. I think before I open eyes, I'm aware that I'm conscious”* (P04, G) Others described it as a behavioural state: *“It's the start of activity isn't it? The start of you know, just realising that you need to get up off the bed”* (P10, G).

When questioned about their sleep-wake transitions and identifying the moment of awakening during the CAR studies, retrospective narratives tended towards being confused and uncertain: *“And you kind of wonder to yourself ‘Have I actually been to sleep, or have I, you know, have I just been sort of laying here in a deep state of rest, but not quite asleep’; It just felt like that, really”.* (P19, A). This was not the only instance of self-dialogue being required to clarify their state of wakefulness. The hazy zone between wakefulness and sleep was also common amongst participants' accounts: *“It wasn't entirely clear-cut, because some mornings you … you kind of surface slowly, don't you? It's a bit ‘foggy’ … It's a progressive event, rather than ‘asleep – awake’”* (P14, A). This description of the sleep-wake transition as a process which may differ across days was prevalent in the narratives, with some participants describing a process of emerging from a dream state into wakefulness: *“I sort of hear the radio in the background and sometimes it merges with the dream because I dream about something similar. ‘There's something strange there … probably this is the radio’ … it is similar to a transitional period. Eventually you do wake up and you realise what you were dreaming about …. then you realise you're conscious”* (P03, G). In general, there was no clear sense of the exact moment of awakening: *“I've never really had – on any of the four occasions, in this study – a clear point at which I could say I was asleep and then awake” (*P19, A). This meant that there was much uncertainty expressed about whether the first morning sample was taken on time illustrated by internal dialogues, for example, “*I was kind of going ‘Right, hang on a minute; what am I going to do; shall I go with this?”* (P15, A).

## 4 Discussion

Findings indicated that participants were motivated to volunteer for altruistic reasons and perceived convenience of participation. However, they experienced the process as somewhat onerous in terms of increased study-specific apprehension and cognitive demand accompanied by disturbed sleep, earlier awakening, and disrupted morning routines. However, this was mostly attributed to the first day of the two sampling days. In general, burden was lessened with repeated sampling days. Participants expressed uncertainty about the understanding and identification of the moment of awakening.

Extrinsic motivations for participating in CAR research were that it was non-invasive and could be conducted in one's home. This corresponds with previous research indicating that the nature of the research is important in contemplating participation [19] and that participants value convenience [20]. More significantly, participants expressed intrinsic altruistic motivations consistent with previous literature suggesting that altruistic motivations are commonly held in research participation [19,[21], [22], [23]]. The importance of intrinsically motivated participation cannot be understated in CAR research for several reasons. Firstly, these studies can be burdensome for participants (see discussion below). Secondly, the opportunity to benefit directly is the primary motive in research participation [21] however, in CAR research, the potential for most participants to benefit personally is limited as there are usually no therapeutic or financial benefits in taking part. Further, CAR studies rely upon finely timed saliva sampling in the immediate post-awakening period in the domestic setting, unsupervised by the researcher, which means that participant accuracy is essential for the quality of the data obtained. Data derived from the current study and others [21] suggest that altruistic motivation can be fostered by demonstrating to the participants that the research is valuable, that participation is worthwhile, for example, by how it could potentially benefit others including implications for clinical groups and contribution to scientific knowledge. Intentional attention to these factors by researchers is recommended [22,23]. In accordance with Moorcraft and colleagues [24] and the CAR consensus guidelines [11,12] researchers aimed to promote and maintain a positive attitude with the participant from the outset. Indeed, the altruism explicitly expressed towards CAR research and researchers in the current study suggests that the participant-researcher relationship is key to fostering the levels of motivation and subsequent conscientious participation.

Whilst motivations were positive and encouraging, participant narratives revealed the somewhat demanding nature of participation. CAR research in the domestic setting is credited with providing ‘ecological validity’ [11,12] as it can be done whilst participants undergo their typical routines, unlike that of sleep laboratory studies. However, participants reported disruptions to their morning routines, particularly on the first sampling days. For instance, participants described their routines as being modified or even postponed so that they could focus on the timing of the saliva samples. More significantly, CAR research participation impacted thoughts, emotions and behaviours. This is an important issue as on a particular day the CAR is determined to a greater extent by state/situational variables, such as mood, cognitive and sleep-related variables, than by more stable trait characteristics [10,25,26]. For example, evidence indicates that prior day mood, anticipations of a more demanding day ahead and prospective memory load are associated with the CAR [9,[27], [28], [29], [30]]. In the current study, participants described the CAR protocol as relatively demanding, with increased anticipatory apprehension, cognitive burden, heightened alertness and the need to be mentally prepared. Further, there were consistencies in the accounts regarding disruptions to sleep, particularly in relation to premature awakening. Early awakening has been found to be associated with a heightened CAR [11], whereas the impact of disturbed sleep on the CAR is less clear. Experimentally induced sleep restriction was found to have no effect on the CAR [31]. However, in a recent study, severe experimental fragmentation of sleep (repeated awakening every 15 min) elicited decreased CARs [32]. The interventional nature of the CAR study protocol appeared to be more prominent on the first day of sampling and was mitigated by repetition.

Participants explained that as they became more familiar with the study protocol there were improvements in sleep quality, wake time, cognitive load and mood in latter study days. Thus, these findings add qualitative support to the CAR consensus guidelines regarding the limited utility of measuring the CAR on a single day [11,12]. It is recommended to measure CAR on at least two study days; however, based on participant reports of decreased burden and ease over repeated sampling days, researchers could extend this to three days with the first day being a ‘practice’ day for participants and the two latter days being test days.

Whilst CAR researchers promote their research on the basis that it is conducted from the comfort of one's home and to fit in with one's routine, they do not usually discuss the potentially disruptive nature of the study. Researchers in the field should be aware of the potentially burdensome nature of CAR research and ensure participants are fully informed prior to participation and or give opportunity for a practice sampling day. This might be particularly important for those who are more vulnerable and those with sleep problems.

Accurate CAR measurement requires participants to identify and act upon the ‘moment of awakening’. Findings from the current study suggest that participants found it difficult to both define and identify awakening. Awakening had different meaning for different participants. For some awakening was defined as a state of consciousness, in line with the definition in the consensus paper [11] and with the explanations given by the CAR researchers to those participants in the current study. For others though awakening was defined as a behavioural state, which is indeed what is generally measured by objective measures of waking such as activity watches. Narratives indicated no clear sense of the exact moment of awakening and descriptions corresponded to a reduced state of cognition resultant from sleep inertia, the neurobehavioural impairments following awakening most marked in higher order processes necessary for cognitive tasks [13,33]. The inability to identify awakening may contribute to sampling inaccuracy by increasing delay in initiating saliva sampling, impacting CAR measurement [11,12,15]. Given their expressed motivation, it is likely that any CAR data measured from inaccurate samples provided by participants interviewed in the current study is due to sleep inertia rather than non-conscientious participant behaviour. Sampling inaccuracy is mostly attributed to collection of the first ‘awakening’ sample [34]. However, when there are accurately measured short delays in sampling (up to 15 min) the post-awakening cortisol curve can still be accurately modelled using real-time data analysis [34]. Thus, to reduce worry and concern about getting it right, participants should be reassured that such short delays between awakening and collection of the first sample are acceptable. Similarly, to reassure participants it is recommended to discuss the common experience of ambiguity about the moment of awakening (see Table 3).

**Table 3:** Methodological recommendations for future CAR research.

Recommendation Comment	
Foster the participant-researcher relationship	Maximise altruistic motivation to adhere to the study protocol by explaining the purpose and value of the study
Repetition of data collection, ideally with initial practice day	Familiarity with the study protocol promotes improvements in sleep quality, and reductions in apprehension and study-specific cognitive load

Inform participants that short delays (up to 15min) are acceptable if accurate awakening and sampling times are recorded, and real-time data analysis used [15]	Reduction in participation apprehension and cognitive load

Discuss the ambiguity of ‘the moment of awakening’	Reduce concern and worry by acknowledging the common experience of difficulty identifying the moment of awakening

The limitations of the study include the reliance on the participants to recall past experiences without biases implicit within any qualitative study. Further, participant perceptions are limited to mostly female volunteer groups from the academic community and those with primary open-angle glaucoma, which may explain the expressed high levels of motivation and limit generalisability of the findings to other populations. Motivations and experiences of other populations are unknown and could be explored in future research. It is worth noting, however, that there was little difference in the experiences expressed, the median time of morning awakening and adherence by the two samples in the current study. The average age of the participants was relatively old (61 years) and awakening time was consistent and moderately early. Morning routines are likely to be different in other, e.g. younger, populations with subsequent effects on the experience of participation in CAR research. For example, some groups may have more erratic or rushed morning routines, the impact of which has not been captured here. Furthermore, these participants were largely healthy, experiences of participation may be different in clinical samples.

This is the first study to qualitatively explore the motivations and experiences of volunteers participating in CAR research in the domestic setting and to explore the subjective meaning of the moment of morning awakening. These participant narratives may be informative for any researcher measuring the cortisol awakening response. The findings highlight the importance of motivated participants given the potentially burdensome nature of CAR research participation. Further, participants reported that participation in CAR research impacted mood, cognition and sleep, state variables known to influence the CAR. Study-specific apprehension and cognitive burden as well as earlier awakening and disruption of routines threaten its ‘ecological validity’ potentially impacting typical CAR characteristics. Repetition of data collection over as many study days as practically possible is recommended as there was an indication of participants experiencing habituation to protocol. Participants struggled to identify the ‘moment of awakening’ whilst experiencing sleep inertia. Researchers need to use electronic measures of awakening to ease apprehension around collecting saliva samples ‘on awakening’. Moreover, researchers may discuss with participants the ambiguity surrounding awakening to increase awareness, lessen anxiety and highlight its importance (see Table 3 below for methodological suggestions).

## Funding source

This research did not receive any specific grant from funding agencies in the public, commercial, or not-for-profit sectors.

## CRediT authorship contribution statement

**Natasha Ramachandran:** Writing – review & editing, Writing – original draft, Methodology, Investigation, Formal analysis, Data curation. **Nina Smyth:** Writing – review & editing, Supervision, Conceptualization. **Sanjay Joban:** Methodology, Formal analysis, Conceptualization. **Maria Flynn:** Writing – review & editing, Supervision. **Angela Clow:** Writing – review & editing, Supervision, Conceptualization. **Lisa Thorn:** Writing – original draft, Supervision, Methodology, Investigation, Formal analysis.

## Declaration of competing interest

The authors declare the following financial interests/personal relationships which may be considered as potential competing interests: Natasha Ramachandran reports article publishing charges was provided by University of Westminster. Natasha Ramachandran reports a relationship with University of Westminster that includes: employment. If there are other authors, they declare that they have no known competing financial interests or personal relationships that could have appeared to influence the work reported in this paper.
